# A *de novo PUM1* Variant in a Girl With a Dravet-Like Syndrome: Case Report and Literature Review

**DOI:** 10.3389/fped.2022.759889

**Published:** 2022-03-21

**Authors:** Yuanzhen Ye, Zhanqi Hu, Jiahui Mai, Li Chen, Dezhi Cao, Jianxiang Liao, Jing Duan

**Affiliations:** Department of Neurology, Shenzhen Children's Hospital, Shenzhen, China

**Keywords:** *PUM1*, Dravet syndrome, epilepsy, developmental disability, ataxia, ptosis

## Abstract

In the recent 3 years, subjects with Pumilio1-associated developmental disability, ataxia, and seizure syndrome have been identified as harboring Pumilio homolog 1 (*PUM1*) mutations. However, the characteristics of the seizure phenotype remain to be elucidated. We herein described a 3-year-old female proband who was diagnosed with developmental and epileptic encephalopathy presenting with some features suggestive of a Dravet-like syndrome. For genetic analyses, trio-based whole-exome sequencing and array comparative genomic hybridization were performed. Consequently, a *de novo* heterozygous missense variant was identified in exon 22 of the *PUM1* gene: NM_001020658: c.3439C > T (p.Arg1147Trp). Upon thoroughly reviewing the existing literature, nine cases of *PUM1* mutation-related epilepsy were identified, and their clinical features were summarized. A relationship between *PUM1* mutation and clinical manifestations characteristic of a Dravet-like syndrome was proposed. To our knowledge, this is the first report of a patient with *PUM1* mutation presenting with a Dravet-like syndrome.

## Introduction

The RNA-binding protein Pumilio homolog 1 (*PUM1*) is a member of the Pumilio family. It binds to specific mRNAs (ATXN1, E2F3, CDKN1B, SAE1, CDK1, and AAMP) and directly prevents its translation, thus negatively regulating the expression of the target genes (1). A loss-of-function mutation of *PUM1* increases the *ATXN1* mRNA level, which leads to Atxn1 accumulation in Purkinje cells located in the cerebellar cortex of the brain, consequently leading to motor dysfunction (2–4), which is responsible for spinocerebellar ataxia type 1 (OMIM: #164400). In recent years, it has been reported that the *PUM1* gene causes a novel developmental disorder called Pumilio1-associated developmental disability, ataxia, and seizure syndrome (PADDAS) (5), which suggests that other brain areas besides the cerebellar cortex are also affected by *PUM1* mutation. The epileptic phenotype remains to be clarified as a characteristic of this syndrome.

Dravet syndrome (DS) is a rare, genetically inherited, epileptic encephalopathy. Epilepticus and status epilepticus triggered by fever are important characteristics of DS. More than 85% of the cases of DS are due to an *SCN1A* mutation. Many other genes have been reported to be associated with DS or Dravet-like syndrome, such as *SCN2A, SCN9A, SCN8A, PCDH19, SCN1B, GABRG2, GABRA1, HCN1, STXBP1, KCNA2*, and *CHD2* (6). However, each gene has its own phenotypic characteristics, not all of which are indicative of typical DS.

Herein, we described a rare case of a patient with developmental and epileptic encephalopathy who presented with some features of Dravet syndrome (DS) and had a *de novo* heterozygous missense mutation in the *PUM1* gene (NM_001020658: c.3439C > T, p.Arg1147Trp). To our knowledge, thus far, only nine patients with epilepsy caused by the variant of the *PUM1* gene have been reported, including this case. Herein, we report what we believe is the first report of a case with a potential phenotype–gene correlation of developmental and epileptic encephalopathy with this novel *PUM1* variant, presenting with characteristic features of a Dravet-like syndrome. Furthermore, we present a literature review of the nine cases identified as having epilepsy linked to *PUM1* variants.

## Materials and Methods

Written informed consent was obtained from the parents of the patient for publication of this case report and the use of any accompanying images in the report. After obtaining informed consent, peripheral venous blood samples were taken from the proband and her parents.

All steps of trio-based whole-exome sequencing (WES) were performed by AmCare Genomics Lab (Guangzhou, China), including genomic DNA extraction, exome library preparation, exome capture, sequencing, and data analysis. Whole-exome libraries were generated using SureSelect Human All Exon V6 Kit (Agilent, Santa Clara, CA). Illumina platform with PE 150 (Illumina, San Diego, CA) was used to perform subsequent sequencing. Sequencing reads were mapped to the reference hg19 version of the human genome.

Patient's chromosomal microarray analysis was performed using Affymetrix Cytoscan HD, with the hybridization and labeling steps performed as per the manufacturer's instructions. Then, Affymetrix Chromosome Analysis Suite 4.2 Software was used to analyze raw data of chromosomal microarray analysis.

## Results

The proband is a 3-year-old girl; she is the second child of non-consanguineous, healthy Chinese parents. Their elder son is healthy, and there is no remarkable medical history of the family. At the time of the birth of the proband, her father and mother were 29 and 28 years old, respectively. She was born at full term with normal delivery. She had normal birth weight (2,950 g), height (49 cm), and occipitofrontal circumference (33 cm).

At 3 months of age, she could hold her head without support. At 4 months, she was able to follow faces and could recognize her mother. At 4 months of age, she experienced an episode of status epilepticus that was triggered by fever every time. The episode continued for ~2 h and was stopped by midazolam administration at the local community hospital. She was then transferred to our hospital for the treatment of status epilepticus. It was observed that during the seizures, her eyes were fixated in a one-sided stare, the right side of her mouth twitched, her right limbs showed stiffening and clonus, and she lost consciousness. These findings persisted for the entire duration of the seizure (2 h). After this seizure, she was reported to have several seizures with a frequency of once per 1–3 months. All these seizures were fever triggered and presented as status epilepticus. She was successively administered courses of levetiracetam (LEV; max dosage at 55 mg/kg.day), valproate (VPA; max dosage at 38 mg/kg.day), and topiramate (TPM; max dosage at 5.2 mg/kg.day); however, no improvement was noted. Perampanel (PER; final dose at 3 mg/day) was added to the combination treatment of LEV, VPA, and TPM at 1 year and 10 months of age, which reduced the frequency of fever-triggered seizures from once a month to only one episode in the last year. It further reduced the seizure duration to 2–3 min. Her development was normal before 4 months of age (held head steady without support and recognized familiar people), and developmental regression was noted after the onset of epilepsy. She started establishing eye contact at 1 year old again and rolling her body at 1 year and 6 months old. She was identified as having profound axial hypotonia and could no longer hold her head after the onset of epilepsy. At the last follow-up when she was 3 years old, gradual developmental improvement was noted, but she had still not begun verbal communication.

**Figure 1:**
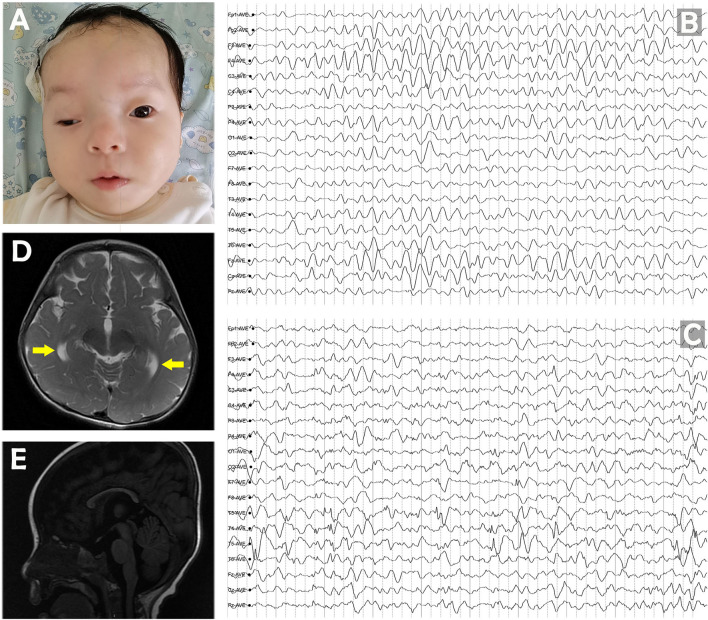
**(A)** Pictures of the patient at the age of 11 months. **(B)** The diffuse slowing background of EEG at 4 months of age. **(C)** Sporadic multifocal spikes of interictal discharge at 11 months of age. **(D)** Brain MRI at 11 months showed that the bilateral temporal horn of the ventricle was enlarged at T2 axial section. **(E)** Sagittal T1 section shows a thin corpus callosum.

She had several dysmorphic features with high-arched palate, ptosis, hypertelorism, broad nasal bridge, low-set ears, bitemporal narrowing, and almond-shaped eyes (Figure 1A). Considering the severe motor delay and hypotonia, she could not be evaluated for ataxia. The background EEG showed diffuse slowing activity initially at 4 months of age. Sporadic multifocal spikes appeared over time in several electroencephalograms taken at different time points (at age ≥11 months), with spike-and-wave discharges noted primarily in the left posterior region of the head. Episodes of focal slow waves were noted in areas of the right parietal, frontal, and temporal lobes (Figures 1B,C). At 11 months of age, bilateral temporal horn of the ventricle was found to be enlarged on brain MRI. Furthermore, a thin corpus callosum was seen (Figures 1D,E), indicating that the white matter may also be affected.

During her last follow-up at 3 years of age, LEV was being tapered, and the other anti-seizure medicines were continued. With these medicines, the seizure frequency had been reduced to once every 5–6 months, and the seizure duration had been reduced to 2–3 min. After the reduction of seizure duration and frequency, no signs of developmental regression were noted. She could now roll over her body and smile at an acquaintance.

Trio-based WES and array comparative genomic hybridization (CGH) were performed. Their findings revealed that the exon 22 of the *PUM1* gene had a *de novo* heterozygous missense variant [NM_001020658: c.3439C > T (p.Arg1147Trp)], which is otherwise absent in population databases (dbSNP, ExAC, and GnomAD). Thus far, at least three unrelated patients with this variant have been reported (5, 7, 8), and all of them were *de novo*. Upon Western blotting analysis, it was found that the *PUM1* protein stability was markedly compromised by this variant (5). Taken together, according to the American College of Medical Genetics and Genomics guidelines, this variant is considered pathogenic (9).

## Discussion

Herein, we described a new patient with a pathogenic variant of the *PUM1* gene and presenting with some characteristic features of a Dravet-like syndrome. She had significant developmental delay and regression as an infant. It is believed that in the classical description of Dravet syndrome, developmental slowing is noted 1 year of age. Nevertheless, in a recent research of Li et al. (10), developmental slowing was noted before 12 months of age in 27% (28/104) patients with Dravet syndrome (range, 3 months to 5 years). Besides, the background EEG activity was initially slow with no abnormal discharge, followed by multifocal discharges over time. According to the International League Against Epilepsy classification of 2017 (11), the background EEG activity is typically normal in the first year of life in DS. In addition, slowing may be seen initially, and diffuse slowing may appear over time (12, 13). Furthermore, her epilepsy presented as fever-triggered and drug-resistant status epilepticus during her infantile period. Therefore, her clinical features share some characteristics of DS, including the course of the disorder, clinical manifestations, and EEG patterns, which we collectively considered as a phenotype indicative of a Dravet-like syndrome. However, she displayed severe developmental delay as an infant, which was atypical for classical DS. Besides epilepsy, she also had dysmorphic features, which is also not characteristic of DS. She is 3 years old at the time of this study and is regularly being followed up. During her most recent follow-up, which was also at the age of 3 years, we noted no verbal communication, severe intellectual disability, and the use of only eye contact to acknowledge other people. Developmental regression could be observed after the onset of epilepsy, and improvement was noted after the seizure episodes reduced, which indicated the diagnosis of developmental and epileptic encephalopathy. The results of trio-based WES indicated that she had a pathogenetic variant of the *PUM1* gene with a *de novo* missense mutation (c.3439C > T, p.Arg1147Trp); this finding has been reported thus far in only three unrelated patients with PADDAS. Until the time of this study, this variant of *PUM1* has not been reported to present with the phenotype of DS or a Dravet-like syndrome.

*PUM1* belongs to a well-characterized family of RNA-binding proteins that are involved in various physiological processes (14, 15). *PUM1* regulates transcription by regulating the function and stability of mRNAs of its target (4). *ATXN1*, which is responsible for spinocerebellar ataxia type 1 (OMIM: #164400), is a clinically significant target of *PUM1*. *ATXN1* is negatively regulated by PUM1-promoted transcript degradation. Thus, a loss-of-function mutation of *PUM1* increases the *ATXN1* mRNA level, which leads to Atxn1 accumulation in Purkinje cells located in the cerebellar cortex of the brain, consequently leading to motor dysfunction (2–4). In mice models, progressive cerebellar degeneration was observed as a result of reduced *Pum1* protein, which resulted in limb and gait ataxia along with fine and gross motor impairment (4). In 2018, Gennarino et al. described *PUM1* variants in patients with intellectual disability and in those with congenital spinocerebellar ataxia with adult onset (5).

Peer-reviewed articles were found using PubMed with search terms: “PUM1,” “Dravet syndrome,” and “Spinocerebellar ataxia 47.” At the time of writing, only nine patients (including our patient) have been reported as having epilepsy caused by this variant of *PUM1* (Table 1). This variant can be associated with conditions presenting with epileptic encephalopathy, such as West syndrome (16). The seizure types could be absence seizures, focal seizures, spasms, and tonic–clonic seizures. Even ketogenic diet and polypharmacy treatment could not completely treat seizures in these patients. The patients had early-onset pharmacoresistant epilepsy and global developmental delay.

**Table 1:** Summary of clinical features of nine patients with epilepsy related to the variant of *PUM1*.

**Feature**	**Our case**	**Case 1** **Voet et al. (8)**	**Case Bonnemason-Carrere et al. (7)**	**Subject 11** **Gennarino et al. (5)**	**Case 2** **Voet et al. (8)**	**PMID: 30536491**	**Subject 1 Gennarino et al. (5)**	**Subject 4** **Gennarino et al. (5)**	**Subject 7 Gennarino et al. (5)**
Gender	F	M	F	F	M	M	/	/	M
Variant/CNV	c.3439C > T (p.R1147W)	c.3439C > T (p.R1147W)	c.3439C > T (p.R1147W)	c.3439C > T (p.R1147W)	c.2509C > T (p.R837*)	(28835332–32362980) ×1	(28751378–33588455) ×1	(31442430–31720099) ×1	(31113947–32897001) ×1
Inheritance	*De novo*	*De novo*	*De novo*	*De novo*	*De novo*	*De novo*	ND	*De novo*	ND
Global DD	Yes	Yes	Yes	Yes	Yes	Yes	Yes	Yes	Yes
Seizure type	Focal seizures and status epilepticus	Absence seizures, focal seizures, and status epilepticus	Absence seizures	ND	Absence seizures and tonic–clonic seizures	Infantile spasms	ND	ND	ND
Seizure onset	4 months	In the first year	3 years	5 months	14 months	4 months	ND	ND	ND
Seizure progrosis	Controlled by treatment	Refractory	Controlled by treatment	Worsened over time	Controlled by treatment	Controlled by treatment	ND	ND	ND
Anti-seizure drugs	LEV, VPA, TPM, and PER	Polypharmacy (without details)	VPA and CLB	CBZ, PM, LTG, LEV, CLB, OXC, CBD, and KD	Polypharmacy (without details)	ND	ND	ND	ND
Ataxia	Could not be evaluated	NO	Could not be evaluated	Yes	NO	Could not be evaluated	Yes	Yes	Yes
Hypotonia	Yes	Yes	Yes	Yes	Yes	Yes	ND	ND	ND
Ptosis	Ptosis	ND	Ptosis	Ptosis	ND	ND	ND	ND	ND
Ocular abnormalities	Exotropia	Hypermetropia and exotropia	Astigmatism with esotropia	Cortical visual impairment	ND	ND	ND	Esotropia	ND
EEG	Sporadic multifocal spikes, spike-and-wave discharges primarily in the left posterior head region, and episodes of focal slow waves in the area of right parietal, temporal, and frontal lobes	Slow, poorly organized background activity with sporadic multifocal epileptic activity	Intermittent slow waves in the background pattern and episodes of high-voltage peak waves and slow waves, compatible with primary generalized epilepsy	Poorly organized background slowing and occasional multifocal epileptiform activity	Intermittent slow waves in the background pattern and episodes of high-voltage peak waves and slow waves, compatible with primary generalized epilepsy	Hypsarrythmia patterns	ND	ND	ND
MRI features	Enlarged bilateral temporal horn of the ventricle and thin CC	Hypoplasia corpus callosum and cerebral atrophy	Shortened CC	Enlarged fourth ventricle and shortened CC	Normal	Normal	ND	ND	ND

*EEG, electroencephalogram; F, female; M, male; MRI, magnetic resonance imaging; ND, information not documented; CBZ, carbamazepine; PM, phenobarbitone; LTG, lamotrigine; LEV, levetiracetam; CLB, clobazam; OXC, oxcarbazepine; CBD, cannabidiol; KD; ketogenic diet; /, not applicable; CC, corpus callosum*.

Similar to these patients, our patient also presented with global developmental delay and status epilepticus easily triggered by fever. Her condition improved after combined treatment with LEV, VPA, TPM, and PER. Because of the early onset, severe global developmental delay, and hypotonia, she could not be evaluated for ataxia. The present case is probably the first of its kind with a likely phenotype–gene correlation of this novel *PUM1* variant with a Dravet-like syndrome.

Gennarino et al. showed that *Pum1* is expressed in all brain regions (4). The MRI results of these patients have shown abnormal signals in or atrophy of corpus callosum, cerebrum, and the periventricular region, thus confirming that multiple regions of the brain may be affected. The MRI of our patient performed when she was 11 months old revealed enlarged bilateral temporal horn of the ventricle. Furthermore, in agreement with previous reports of such patients, a thin corpus callosum could be seen in our patient (5, 8). Epilepsy is the dysfunction of the cerebrum (17), and we believe that epilepsy and abnormal cortical EEG findings in these patients may indicate that their cerebrum may also be affected.

Mutation type in *PUM1* included missense mutation, nonsense mutation, and microdeletion. Besides, the presence of a *PUM1* mutational hotspot may be indicated by the findings that four unrelated patients had the same *de novo* variant p.Arg1147Trp. Gennarino et al. showed that this variant was associated with significantly reduced levels of the Pum1 protein by affecting their stability; the levels in patient's fibroblasts were reduced to ~43% of the controls. Conversely, mutations reducing PUM1 levels by 25% lead to a milder phenotype and adult-onset ataxia (5). These findings indicate that the extent of severity of the phenotype differs with the levels of the PUM1 protein. We speculate that in these patients, the variants associated with severe phenotype of epileptic encephalopathy markedly affected the PUM1 protein levels.

Besides epilepsy, these patients also had dysmorphic features, such as almond-shaped eyes, ptosis, high-arched palate, broad nasal bridge, low-set ears, and bitemporal narrowing, which could also be seen in our patient. Taken together, we herein described a new patient with a heterozygous *de novo* c.3439C > T p.Arg1147Trp *PUM1* missense variant who has been suffering from early-onset PADDAS. Furthermore, we reviewed all the patients with seizure. Although we only have a single case report as evidence, our report suggests that the PADDAS phenotype may align with the characteristic presentation of a Dravet-like syndrome. More cases showing similar presentation would be needed to support our findings.

## Data Availability Statement

The raw data supporting the conclusions of this article will be made available by the authors, without undue reservation.

## Ethics Statement

Written informed consent was obtained from the relevant individual(s), and/or minor(s)' legal guardian/next of kin, for the publication of any potentially identifiable images or data included in this article.

## Author Contributions

YY was the first clinician to meet the patient when she was transferred to the hospital and drafted the manuscript along with JD. JD was in charge to interpret the genetic data. ZH and JM participated in case collection. LC, DC, and JL were members of the treatment team of this patient and participated in the revision of the manuscript. All authors have read and approved the final manuscript.

## Funding

This work was supported by the Sanming Project of Medicine in Shenzhen (No. SZSM201812005), Shenzhen Fund for Guangdong Provincial High Level Clinical Key Specialties (No. SZGSP012), Shenzhen Key Medical Discipline Construction Fund (No. SZXK033), and Brain Cognition and Brain Disease Institute Fund (No. NYKFKT20190014). The funding bodies had no role in the design of the study, the collection, analysis, or interpretation of the data, or writing the manuscript.

## Conflict of Interest

The authors declare that the research was conducted in the absence of any commercial or financial relationships that could be construed as a potential conflict of interest.

## Publisher's Note

All claims expressed in this article are solely those of the authors and do not necessarily represent those of their affiliated organizations, or those of the publisher, the editors and the reviewers. Any product that may be evaluated in this article, or claim that may be made by its manufacturer, is not guaranteed or endorsed by the publisher.

## Abbreviations

PADDAS, Pumilio1-associated developmental disability, ataxia: and seizure syndrome
LEV: levetiracetam
VPA: valproate
TPM: topiramate
PER: perampanel
EEG: electroencephalogram
WES: whole-exome sequencing
array CGH: comparative genomic hybridization
RBP: RNA-binding protein.
